# Everything you need to know about distal renal tubular acidosis in autoimmune disease

**DOI:** 10.1007/s00296-014-2993-3

**Published:** 2014-03-29

**Authors:** Tim Both, Robert Zietse, Ewout J. Hoorn, P. Martin van Hagen, Virgil A. S. H. Dalm, Jan A. M. van Laar, Paul L. A. van Daele

**Affiliations:** 1 Division of Clinical Immunology, Department of Internal Medicine, Erasmus Medical Centre, PO Box 2040, Room D-XXX, 3000 CA Rotterdam, The Netherlands; 2 Division of Nephrology and Transplantation, Department of Internal Medicine, Erasmus Medical Centre, Rotterdam, The Netherlands

**Keywords:** Autoimmunity, Renal physiology, Acid–base balance

## Abstract

Renal acid–base homeostasis is a complex process, effectuated by bicarbonate reabsorption and acid secretion. Impairment of urinary acidification is called renal tubular acidosis (RTA). Distal renal tubular acidosis (dRTA) is the most common form of the RTA syndromes. Multiple pathophysiologic mechanisms, each associated with various etiologies, can lead to dRTA. The most important consequence of dRTA is (recurrent) nephrolithiasis. The diagnosis is based on a urinary acidification test. Potassium citrate is the treatment of choice.

## Introduction

Distal renal tubular acidosis (dRTA) is characterized by an impairment of normal urinary acidification process in the distal part of the nephron in the presence of a normal glomerular filtration rate. The term “distal” implies that acidification by the distal parts of the nephron (connecting tubule and collecting duct) are disturbed in contrast to proximal tubular acidosis, in which the reabsorption of bicarbonate by the proximal tubule is impaired. The prevalence and incidence of dRTA in the population are not known. dRTA is associated with autoimmune diseases such as primary Sjögren syndrome and systemic lupus erythematosus [1–3]. Prevalence of dRTA in primary Sjögren syndrome is estimated to be 5–25 % [4–7]. Recurrent nephrolithiasis and/or chronic metabolic acidosis with a randomly measured high urinary pH suggest the presence of dRTA. Of patients with dRTA, approximately 5 % develops nephrolithiasis (mainly calcium phosphate stones), while 56 % of dRTA patients has significant nephrocalcinosis [8, 9]. Vice versa, in 41 % of the patients with calcium phosphate stones, dRTA is the underlying condition [10]. The availability of an effective treatment for dRTA should lower the threshold for testing suspected patients [11, 12]. To confirm the diagnosis of dRTA, an urinary acidification test is recommended using either the well-known ammonium chloride test or a recently proposed combination of furosemide and fludrocortisone [13].

The aim of this review is to make physicians aware of a disorder in urinary acidification in patients presenting with a chronic metabolic acidosis and/or nephrolithiasis, especially in case of calcium phosphate stones. Both the physiology of renal acid–base regulation and the clinical aspects of dRTA will be reviewed.

## Acid–base homeostasis

Our basal metabolic reactions and daily food intake lead to acid excess. Carbon dioxide (CO_2_) originating from the oxidation of carbohydrates, fats, amino acids and proteins is by far the largest potential source of acid (15.000 mmol/day). CO_2_ is a volatile acid that is removed by pulmonary ventilation, preventing CO_2_ to react with H_2_O to form protons [14].

Human metabolism also produces nonvolatile acids (e.g., phosphate, sulfate) and nonvolatile bases (e.g., bicarbonate), which cannot be excreted by the lungs. Together with acid from our diet and intestinal base loss, the body is exposed to approximately 70–100 mmol of nonvolatile acids per day [15]. The role of the kidney is to excrete this acid excess as well as to monitor arterial pH to maintain a normal acid–base balance.

The kidney can maintain the arterial pH between 7.35 and 7.45 by preventing loss of filtered bicarbonate (4,320 mmol/day HCO_3_^−^) and by net secretion of H^+^ (70–100 mmol/day). The kidney cannot simply secrete this amount of acid, because this would require urinary pH to decrease to approximately 1.3. Due to the energetic maximum of H^+^-ATPase, urinary pH can be maximally decreased to 4.2, which is not sufficient to clear the acid excess [16]. In order to get rid of the acid excess, secreted protons will (1) be titrated by filtered bicarbonate, resulting in bicarbonate reabsorption, (2) excreted by titratable acids, (3) titrated and excreted by ammonium and (4) excretion of free protons.

### Proton secretion

The secretion of protons over the apical membrane is for 90 % achieved by the so-called Na^+^-H^+^ exchanger isoform 3 (NHE3) that exchanges sodium for protons over the apical membrane. This transporter is present in the proximal tubule, thick ascending limb and distal convoluted tubule and is dependent on the basolateral Na^+^/K^+^ pump activity [16]. A second mechanism to secrete protons is carried out by the vacuolar H^+^-ATPase located in the distal tubule (10 %). The vacuolar H^+^-ATPase is limited to create a chemical gradient of 10^3^ of H^+^ over the apical membrane. This limitation is caused by a lack of ATP to keep the transporter functioning at a higher gradient. The maximally reached gradient over the apical membrane is reflected by a decrease in urinary pH from 7.5 to 4.5 [17].

### Titration of bicarbonate

The kidney filters about 4,320 mmol/day of bicarbonate, of which 99.9 % is reabsorbed [16]. The proximal convoluted tubule is responsible for the reabsorption of 80–85 % of filtered HCO_3_^−^ [18]. Remaining HCO_3_^−^ is reabsorbed further downstream in the nephron. All intraluminal bicarbonate can be protonated and subsequently reabsorbed. This means that the complete reabsorption of filtered HCO_3_^−^ requires 4320 mmol/day of secreted protons, which is considerably more than the 70–100 mmol/day of proton secretion required for neutralizing of nonvolatile acids. However, the process of HCO_3_^−^ reabsorption is not accompanied by net H^+^ excretion.

### Titratable acid excretion

Secreted protons will also interact with buffers other than HCO_3_^−^. These buffers originate from metabolic reactions. The most significant buffers are phosphate (pKa = 6.8), urate (pKa = 5.8) and creatinine (pKa = 5.0) [16]. With a lower urinary pH, a higher percentage of the buffer will be protonated, regardless of the pKa of each buffer.

In the proximal convoluted tubule are the so-called sodium-phosphate cotransporters (NaPi) located that are responsible for phosphate reabsorption. Early studies already showed that these transporters are down-regulated in periods of metabolic acidosis [19]. Recent studies indicate that these transporters are directly inhibited by protons, resulting in hyperphosphaturia [20]. Because of its relative high pKa and the pH-dependent reabsorption of phosphate, phosphate is an important buffer.

The amount of buffer that is ultimately excreted in the urine is largely dependent on the GFR and the plasma concentration of the buffer. For example, an average individual with a normal plasma phosphate concentration and normal GFR will excrete approximately 30 mmol/day of phosphate.

### Regulation of ammonia secretion

**Fig. 1:**
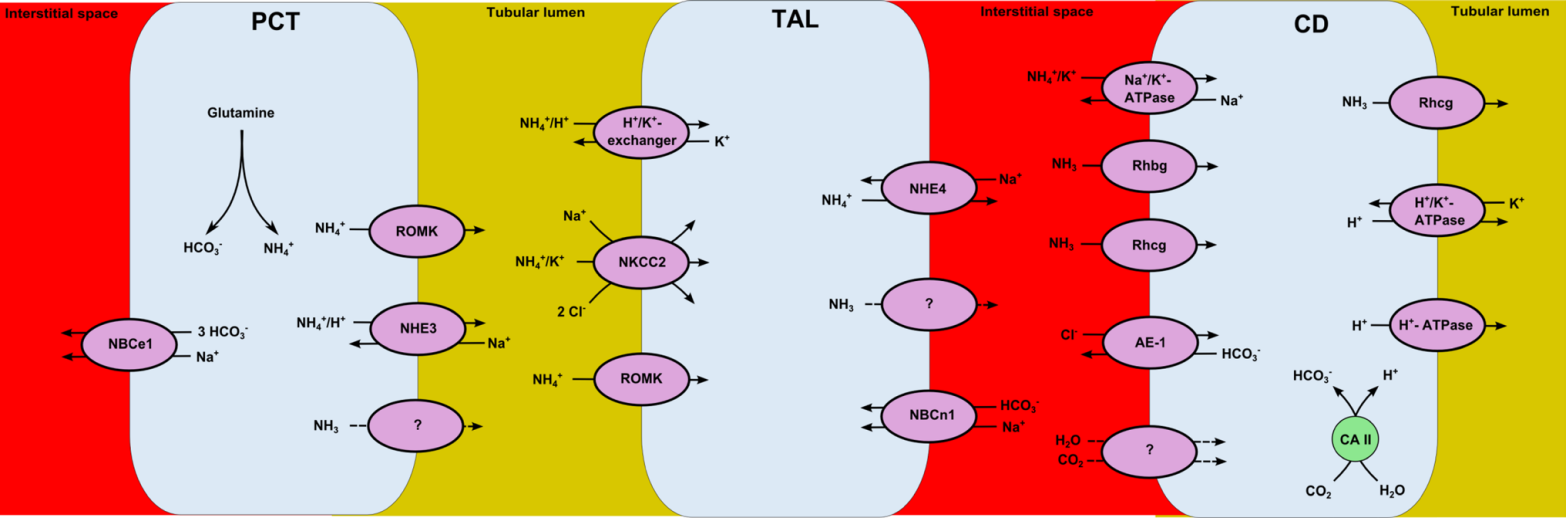
Overview of ammonium transport through the nephron. Ammoniagenesis takes place in the proximal convoluted tubule cells and ammonium is subsequently secreted. The thick ascending limb reabsorbs intraluminal ammonia in order to create a chemical gradient. The collecting duct utilizes this gradient to secrete ammonia over the apical membrane to buffer the simultaneously secreted protons. PCT, proximal convoluted tubule; TAL, thick ascending limb; CD, collecting duct; NBCe-1, Na^+^-HCO_3_^−^ cotransporter; NHE-3, Na^+^-H^+^ exchanger isoform 3; ROMK, Ba^2+^-sensitive K^+^ channel; NKCC2, Na^+^-K^+^-(2Cl^−^) cotransporter; NBCn-1, sodium-bicarbonate cotransporter; Rhbg, Rhesus glycoprotein type B; Rhcg, Rhesus glycoprotein type C; AE-1, chloride-bicarbonate cotransporter

Ammonia (NH_3_) is extremely important as urinary buffer, because of its high pKa of 9, which means that almost all the ammonia will be protonated to ammonium (NH_4_^+^). NH_4_^+^ is in equilibrium with NH_3_ and H^+^ in both the intra- and extracellular space of the nephron. Ammonia is produced in every segment of the nephron, but predominantly in the proximal tubule by the metabolism of mitochondrial glutamine (Fig. 1) [21]. Produced ammonium is secreted by the proximal tubule by NHE3-mediated Na^+^/H^+^ exchange and Ba^2+^-sensitive K^+^ channels (ROMK) [22, 23]. Additionally, NH_3_ is transported over the apical membrane by still undefined channels. Secreted ammonium will be reabsorbed in the thick ascending limb of Henle’s loop either via the K^+^/H^+^(NH_4_^+^) exchanger, or by the Ba^2+^-sensitive K^+^ channels (ROMK) or by the Na^+^-K^+^-(2Cl^−^) cotransporter (NKCC2) [24]. Electroneutral K^+^/NH_4_^+^ exchange and diffusive NH_3_ transport across the apical plasma membrane by undefined channels take also place, but are less important. Cytosolic NH_4_^+^ will mainly exit the tubulus cell via the basolateral NHE4 transporter [25]. A second mechanism of basolateral NH_4_^+^ exit may involve dissociation of NH_4_^+^ to NH_3_ and H^+^. Transport of NH_3_ over the basolateral membrane in the thick ascending limb is presumed to be via diffusion as evidence for a gas transporter for NH_3_ in the thick ascending limb is lacking. However, the concept that gasses (NH_3_ and CO_2_) and water diffuse over the membranes has been questioned over the last years. Instead of diffusion, gasses and water are carried over the membrane by transporters, such as aquaporins and the recently discovered rhesus glycoproteins [26].

The thick ascending limb buffers intracellular produced protons via basolateral bicarbonate transport. This is mediated by the sodium-bicarbonate cotransporter (NBCn1) leading to the formation of H_2_CO_3_ [27]. H_2_CO_3_ will be dissociated into H_2_O and CO_2_, after which CO_2_ will be transported over the basolateral membrane into the peritubular lumen.

Ammonium in the peritubular space will be transported in the collecting duct via Na^+^-K^+^-ATPase and Rhesus glycoproteins Rhbg and Rhcg [26, 28]. Intracellular ammonia will be secreted over the apical membrane via the Rhcg glycoprotein and becomes available to buffer secreted protons [26]. Formed ammonium in the collecting tubular lumen is trapped and will be excreted.

The complex system of ammonia transport through the nephron provides the collecting tubule a chemical and concentration gradient over the apical membrane. By altering these gradients, ammonia secretion over the apical membrane in the collecting tubule can be regulated to buffer the secreted protons.

## Proximal acidification

**Fig. 2:**
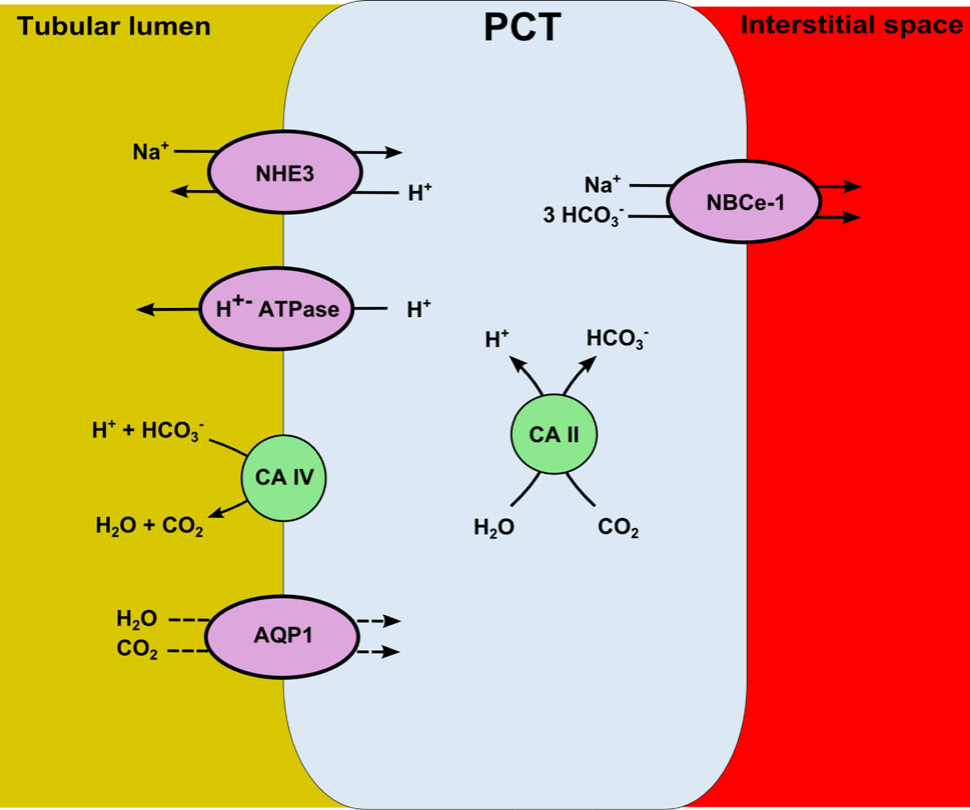
Process of bicarbonate reabsorption in the proximal tubule cell. Filtered bicarbonate is catalyzed by carbonic anhydrase type 4 into carbon dioxide and hydroxide. Carbon dioxide is transported over the apical membrane via aquaporin 1 (AQP1) in the proximal tubule, after which it hydrates into H_2_CO_3_. This reaction is catalyzed by intracellular carbonic anhydrase type 2. Intracellular formed bicarbonate will leave the cell via the NBCe-1 transporter localized on the basolateral membrane. PCT, proximal convoluted tubule cell; CA, carbonic anhydrase; NHE-3, Na^+^-H^+^ exchanger isoform 3; NBCe-1, Na^+^-HCO_3_^−^ cotransporter

As described before, reabsorption of bicarbonate is mainly achieved by proximal convoluted tubule cells (Fig. 2). Secreted H^+^ binds to HCO_3_^−^ to form carbonic acid (H_2_CO_3_) in the tubular lumen. Subsequently, formed H_2_CO_3_ will become H_2_O and CO_2_, a reaction catalyzed by the membrane-bound enzyme carbonic anhydrase type 4.

Luminal CO_2_ and H_2_O are transported over the apical membrane via aquaporin 1 (AQP1) in the proximal tubule, after which they hydrate into H_2_CO_3_. This reaction is catalyzed by intracellular carbonic anhydrase type 2 (CAII). Intracellular H_2_CO_3_ ionizes to H^+^ and HCO_3_^−^, after which HCO_3_^−^ will be transported over the basolateral membrane via the Na^+^-HCO_3_^−^ cotransporter (NBCe-1) [29]. Protons remain in the cytoplasmatic compartment to be secreted again in the tubular lumen. At the end, this process results in the reabsorption of one molecule HCO_3_^−^ and zero net secretion of one molecule of H^+^.

## Distal acidification

**Fig. 3:**
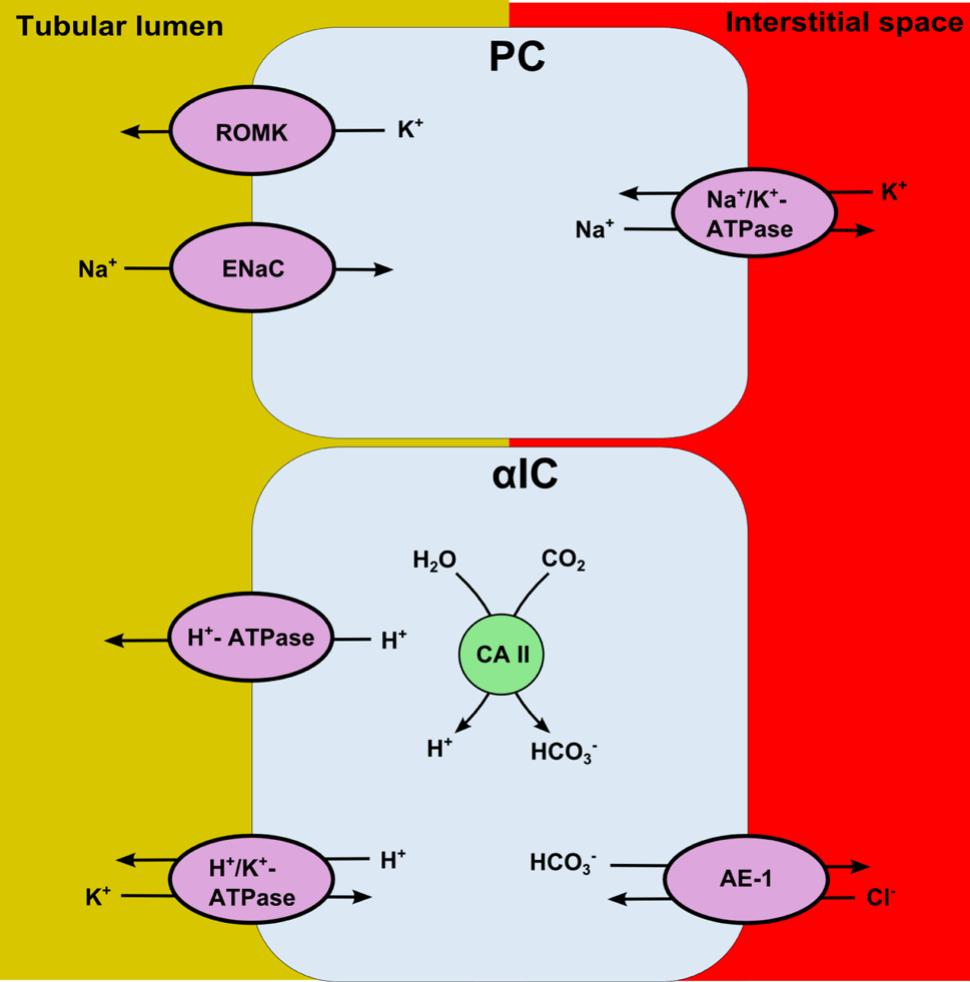
Process of proton secretion in the collecting duct. The principal cell reabsorbs intraluminal sodium creating an electronegative gradient. The alpha-intercalated cells contain vacuoles which stores H^+^ ATPases. These proton pumps are built in the apical membrane for proton secretion. The secretion of protons is enhanced by sodium reabsorption and an electrical gradient. PC, principal cell; αIC, alpha-intercalated cell; CA, carbonic anhydrase; ROMK, renal outer medullary potassium channel; ENaC, epithelium Na^+^ channel; AE-1, chloride-bicarbonate cotransporter

The α-intercalated and principal cells, located in the collecting tubule, are responsible for the secretion of protons (Fig. 3). The principal cell’s main function is to reabsorb sodium via the epithelium Na^+^ channel (ENaC) located in the apical membrane [30]. This causes an electronegative tubular lumen, favoring the secretion of potassium or protons.

Proton secretion is achieved by the vacuolar H^+^-ATPase, stored in vacuoles in the cytoplasm of α-intercalated cells. The expression of this pump is largely dependent on the electrical gradient over the luminal membrane. The electronegative luminal potential, driven by ENaC activity, results in expression of H^+^-ATPase on the apical membrane of the α-intercalated cells and excretion of protons into the lumen [28]. The protons are generated by intracellular activity of the CAII enzyme, which also forms HCO_3_^−^ ions. HCO_3_^−^ will be exchanged with Cl^−^ over the basolateral membrane via the chloride-bicarbonate cotransporter (AE-1) [28]. Still another ATPase expressed in the apical membrane of the α-intercalated cell is the H^+^/K^+^ exchanger. This exchanger contributes to proton secretion, but is less important than the vacuolar H^+^-ATPase and is considered to be more relevant for potassium reabsorption.

## Distal renal tubular acidosis

The characteristic features of dRTA are the presence of systemic acidosis together with the inability to acidify the urine to a pH <5.3 dRTA is associated with many diseases each with their own pathophysiology. To provide a clear overview of the causes of dRTA, we divided dRTA into four groups based on their pathophysiologic defect: (1) voltage defect, (2) H^+^ secretion defect, (3) H^+^ gradient defect and (4) ammonium generation defect (Table 1).

**Table 1:**
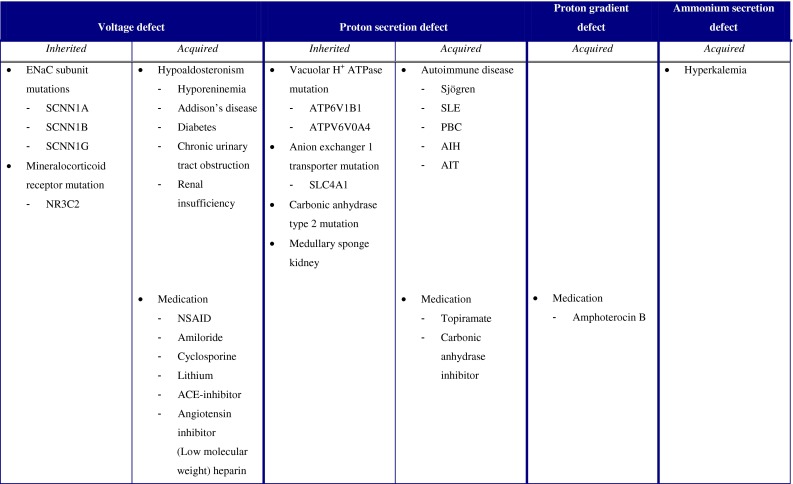
Overview of the aetiology of renal tubular acidosis

### Voltage defect

As outlined before, an electronegative luminal potential in the collecting tubule contributes to proton secretion. The ENaC is responsible for this driving force by reabsorbing Na^+^. ENaC’s activity is predominantly regulated by aldosterone. Apart from regulation of ENaC activity aldosterone can enhance distal urinary acidification by increasing the activity of H^+^-ATPase in the cortical collecting tubule [30, 31].

Both genetic and acquired forms of decreased ENaC activity exist. Genetic causes are related to mutations in genes encoding for the alpha, beta or gamma subunit of the channel (respectively, SCNN1A, SCNN1B, SCNN1G genes), resulting in autosomal recessive pseudohypoaldosteronism type 1. An autosomal dominant form in which the genetic defect (NR3C2) affects the mineralocorticoid receptor is also known [32].

Acquired forms of decreased ENaC activity are more common. They are common due to hypoaldosteronism. The most common cause of hypoaldosteronism is hyporeninemia as can occur in diabetes mellitus, renal insufficiency or use of nonsteroidal anti-inflammatory drugs or calcineurin inhibitors [33]. Furthermore, aldosterone is diminished in Addison’s disease. Additionally, medication can directly or indirectly decrease ENaC activity (e.g., amiloride, cyclosporine, tacrolimus, lithium, ACE-inhibitors, angiotensin II receptor blocker, aldosterone receptor blockers and heparin) [32, 33].

### H^+^ secretion defect

Alpha-intercalated cells are responsible for both generation and secretion of protons. The intracellular enzyme CAII catalyzes the reaction leading to the formation of protons and bicarbonate ions. The main proton transporter is the vacuolar H^+^ ATPase, built in the apical membrane. The bicarbonate ion is transported over the basolateral membrane by the AE1. A defect in one of those subparts of the H^+^ secreting machinery can lead to dRTA.

Primary causes for a defect in one of the compartments are due to mutations in genes encoding subunits of the vacuolar H^+^ ATPase (ATP6V1B1 and ATPV6V0A4), resulting in impaired transporter function. These mutations lead to autosomal recessive forms of dRTA that can coexist with and without deafness. Also an autosomal dominant form of dRTA is known, caused by a mutation of a gene coding for the AE1 (SLC4A1), leading to a decreased number of this transporter in the basolateral membrane. Carbonic anhydrase enzyme type 2 deficiency by genetic mutations leads to both proximal and distal RTA [34]. Medullary sponge kidney is also a primary cause of dRTA, related to the malformation of the distal tubules. The presence of dRTA in these patients depends on the number of nephrons affected [35].

Acquired impaired transporter function of the H^+^ secreting machinery is often associated with autoimmune diseases like Sjögren syndrome and SLE. In patients with primary Sjögren syndrome, inhibitory autoantibodies against the CAII enzyme have been reported [36]. Also certain medications, such as topiramate and acetazolamide, can inhibit the function of the CAII enzyme [37].

### H^+^ gradient defect

Proton secretion is dependent on the H^+^ gradient over the apical membrane, which is achieved by vacuolar H^+^ ATPase. Notwithstanding an appropriately working vacuolar H^+^-ATPase, creating of such gradient is not always successful. This is the case in leaky membrane, sometimes seen in patients using amphotericin B [38, 39]. In experimental models, amphotericin B increases the permeability for protons of the apical membrane in the collecting duct, causing back diffusion of the secreted protons [40, 41].

### Ammonium secretion defects (hyperkalemia)

Ammonium plays a major role in renal urinary acidification. In case of low availability of ammonium in urine, urinary acid excretion is impaired to a certain pH. The most important cause of decreased urinary ammonium is hyperkalemia [42]. Hyperkalemia reduces the expression of ammoniagenic enzymes and acid transport proteins [43]. Additionally, hyperkalemia decreases the secretion of ammonia in the loop of Henle and the collecting duct. This probably is due to competition between NH_4_^+^ and potassium. NH_4_^+^ and potassium use the same binding spot on the transporters in the thick ascending limb (respectively, NKCC2 and Na^+^-K^+^-ATPase) [44]. Hyperkalemia will also drive protons from intracellular to extracellular, leading to a decreased concentration of protons in the distal tubule cells.

## Clinical presentation

The most common symptom of dRTA is nephrolithiasis and metabolic acidosis. Fatigue is a frequent complaint, possibly related to the metabolic acidosis-induced hyperventilation. Patients with chronic metabolic acidosis are prone to develop osteoporosis. Metabolic acidosis affects bone by exchanging protons for sodium, potassium, calcium, carbonate and phosphate [45]. The continuous sequestration of protons in bone stimulates both osteoclast development and osteoclast activity. As a consequence bone resorption increases, enhancing release from the bone surface of calcium and mineral buffers like bicarbonate and phosphate [45, 46]. Eventually, this mechanism leads to net bone loss and hypercalciuria.

Metabolic acidosis also leads to enhanced proximal tubular reabsorption of citrate, resulting in hypocitraturia. Alkaline urine in combination with hypocitraturia and hyperphosphaturia promotes calcium phosphate precipitation leading to nephrocalcinosis and/or kidney stones [47].

Additionally, patients with dRTA often develop abnormalities in the potassium balance. In general, metabolic acidosis will lead to hyperkalemia as a result of the exchange of protons for intracellular potassium. However, patients with dRTA due to a proton secretion defect tend to waste potassium in urine in order to maintain electroneutrality over the apical membrane. Despite potassium wasting, these patients usually have normal levels of serum potassium, because of potassium movement from intracellular to extracellular. Nevertheless, case-reports have been described of patients with dRTA who present to the emergency department with hypokalemic paralysis, including respiratory arrest [1, 48].

### Incomplete dRTA

Of the RTA syndromes, also an incomplete form of dRTA is known, including patients with nephrocalcinosis or urolithiasis but without metabolic acidosis. Patients with incomplete dRTA cannot acidify their urine, but a higher amount of NH_4_^+^ excretion compensates for the acid secretion defect. Donnelly et al. hypothesized that this increased NH_4_^+^ excretion originates from an increased production and secretion of ammonium in the proximal convoluted tubule. Additionally, hypocitraturia in these patients is often present. Diagnosis and treatment is the same as for complete dRTA [49].

### Association of dRTA with autoimmune diseases

It is suggested that dRTA is more prevalent in autoimmune diseases. Shearn et al. [50] reported in 1965 the first case of dRTA revealing Sjögren syndrome. Both primary and secondary Sjögren syndrome is associated with dRTA [4, 51–53]. Other autoimmune diseases such as SLE [54], primary biliary cirrhosis (PBC) [55], autoimmune hepatitis (AIH) [56] and autoimmune thyroiditis (AIT) [53] are less common associated with dRTA. The prevalence of dRTA in Sjögren syndrome is currently estimated to be 25 % [4]. The clinical presentation of dRTA in patients with an autoimmune disease is similar to that of those patients without a systemic disease.

The pathophysiological mechanism of dRTA in relation to autoimmunity remains unclear. Several reports suggest that autoantibodies against the CAII enzyme [36, 57] or the acid–base transporters are involved in the pathogenesis of dRTA in autoimmune disease [58]. Recently, Espinosa et al. [59] reported that anti-Ro52 autoantibodies from patients with Sjögren syndrome inhibit Ro52 E3 ligase activity. In vitro inhibition of the ubiquitination process may increase the transcription of pro-inflammatory genes leading to local inflammation and tissue damage [59]. Interstitial inflammation is often found in renal biopsies.

It is unknown whether treatment with corticosteroids in autoimmune disease has a positive effect on dRTA. We advise to treat dRTA in autoimmune diseases with potassium citrate. Potassium citrate is an effective treatment for both the symptoms and complications of dRTA, by restoring acid–base balance (see below). Studies about prognosis of dRTA in autoimmune diseases are lacking.

## Diagnosis

Urinary acidification was assessed using the oral ammonium chloride loading test (NH_4_Cl test). The complete test takes eight hours and does not require blood testing. The test can be unpleasant, because it can induce gastric irritation, nausea and vomiting. Thus, there was room for the development of a quicker and more patient-friendly urinary acidification test. Walsh et al. [13] described in 2007 a urinary acidification test using simultaneous furosemide (40 mg) and fludrocortisone (1 mg) administration. Simultaneous administration of furosemide and fludrocortisone stimulates the kidney to secrete H^+^ ions. Furosemide inhibits the NKCC2 cotransporter, resulting in a higher Na^+^ delivery in the collecting tubule. Fludrocortisone binds and activates the mineralocorticoid receptor in the cytoplasm leading to an increased ENaC activity, thereby enhancing sodium reabsorption and potassium secretion. Additionally, fludrocortisone stimulates the expression of vacuolar H^+^ ATPase in the apical membrane. Increased sodium reabsorption leads to an electronegative luminal potential, which is the driving force for the secretion of protons by the vacuolar H^+^ ATPase in the distal tubule.

Walsh et al. [13] compared this new test to the NH_4_Cl loading test in 10 healthy controls. Every control was capable to acidify their urine to a pH <5.3. The minimum pH value was 4.92 ± 0.10 after furosemide and fludrocortisone administration.

Both tests had the same result of (impaired) urinary acidification in dRTA patients. All patients failed to acidify their urine to a pH <5.3. The lowest measured pH was 6.59 ± 0.13 after furosemide/fludrocortisones administration. The furosemide/fludrocortisone test was better tolerated and lasts shorter it may prefer over the NH_4_Cl test.

## Treatment

The main goal of any treatment for dRTA is to reverse the acidosis, which reduces calciuria and simultaneously increases citrate excretion. This leads to a lower risk of nephrolithiasis and osteoporosis. Currently, potassium citrate (1–2 mEq/kg/day) is the treatment of choice for the management of patients with dRTA. With potassium citrate, not only a bicarbonate donor is provided to treat acidosis, but potassium wasting is compensated simultaneously. Potassium citrate treatment in dRTA patients seems to have positive effects on bone mineral density and bone cell function [11]. Additionally, a recent randomized controlled trial showed that potassium citrate increases bone density and reduced fracture risk in healthy elderly without RTA [12].

## Conclusions

In this review, we discussed the physiology of acid–base homeostasis and translated this mechanism to the RTA syndromes. The pathophysiology is divided into four categories each associated with different etiologies. Physicians should test for dRTA in patients with (recurrent) calcium phosphate stones and/or a chronic metabolic acidosis. The diagnosis of dRTA is made using a urinary acidification test, in which the patient is unable to acidify the urine to pH <5.3. Treatment of dRTA is based on restoring the acid–base balance, which can be achieved with potassium citrate.
